# Role of Strong Opioids in an Effective Discharge for Lower-Limb Large Joint Arthroplasty Patients: A Patient-Based Analysis

**DOI:** 10.7759/cureus.74727

**Published:** 2024-11-29

**Authors:** Raja Muhammad Mussab, Aiman Jawad, Muhammad Tahir Iqbal, Muhammad Awais Iqbal, Prakash Palaparthy, Faris Ali

**Affiliations:** 1 Orthopaedics and Trauma, Jinnah Postgraduate Medical Centre, Karachi, PAK; 2 Orthopaedics and Trauma, Russells Hall Hospital, Dudley, GBR

**Keywords:** analgesic combination, arthroplasty lower limb, eras protocols, opoids, patient satisfaction, post-op pain management, total hip replacement (thr), total knee replacement (tkr), trauma and orthopaedics, who analgesic ladder

## Abstract

Background/objective: Adequate postoperative analgesics are an essential element in the recovery and rehabilitation of large joint lower-limb arthroplasty patients in their acute postoperative phase. In this study, we will establish that strong opioids like morphine should be included as postoperative analgesics to improve patient satisfaction.

Material: This retrospective cross-sectional study was conducted in the Arthroplasty Ward, Trauma, and Orthopaedics Department in a district general hospital of the United Kingdom. Fifty patients operated in January 2024 were enrolled in this study, out of which 25 had total hip replacement and 25 had total knee replacement. Patients were divided into two groups based on analgesics given at the time of discharge. Group A had a strong opioid and Group B had a non-steroidal anti-inflammatory drug (NSAID) plus weak opioids upon discharge. Patients with hospital stays of more than four days and patients with allergies to any analgesics were excluded.

Results: Forty percent (40%) of the patients in the total hip replacement (THR) group and fifty percent (50%) in the total knee replacement (TKR) group were discharged on adequate analgesia (NSAID + weak opioids + strong opioids) and all reported manageable postoperative pain. A significant difference in pain scores on the fifth postoperative day (POD) was observed between the two groups (p = 0.001). Patient satisfaction levels also differed notably between the groups, with significant variance (p = 0.011). Group A showed a higher rate of "very satisfied" patients (n = 3).

Conclusion: Adequate analgesics prescribing is an integral part of enhanced recovery after surgery (ERAS) guidelines for patients undergoing knee and hip arthroplasties. Pain has catabolic systemic consequences for patients and delays postoperative recovery. We have proposed the step ladder pattern of analgesics for such patients, in which strong opioids should be given to aid in pain relief. Apart from this, a virtual consultation should be done by an arthroplasty nurse within one week of operation for their pain assessment as the pain scale.

## Introduction

Total hip and knee replacement or lower-limb large joint replacement arthroplasty is a routine surgical procedure that is carried out on patients with painful conditions such as osteoarthritis and rheumatoid arthritis [1]. As the number of such procedures grows globally, efficient control of postoperative pain becomes necessary to avoid adverse outcomes and improve outcomes [2]. Chronic pain is common after surgery, exacerbates the condition, and slows patients’ recovery progress, physical therapy, and quality of life [3]. Non-selective opioids such as morphine, oxycodone, and hydromorphone are often used for the management of acute postoperative pain since they are potent in severe pain management [4,5]. However, questions have arisen about opioid dependence, side effects, and chronic use leading to a consistent worry on the side of strong opioids for post-discharge treatment. Monitoring and treating pain in patients post-discharge remain a significantly contentious issue due to the potentially complicated usage of opioids and the presence of dependency and tolerance, as well as side effects, such as nausea, sedation, and respiratory depression [4,6,7]. Nevertheless, because they are effective in providing pain relief, they remain a regular part of discharge planning as long as there are no more effective substitutes [8].

A review of evidence specifically on opioid-based analgesia for arthroplasty suggests that opioids are quite useful in relieving the often sharp acute postoperative pain and benefiting early recovery phases [9]. Literature reviews and analyses indicate that patients who receive strong opioids postoperatively experience less pain with significantly lower pain scores during the first 24 hours as it advances to early mobility and physical therapy follow-through [10]. A meta-analysis found that opioids when given in the short term significantly improve pain relief of patients undergoing hip and knee arthroplasty [11]. However, recently, there emerged a concern about increases in the number of patients who depend on opioid drugs to manage their pain [12]. An analysis of available options like non-steroidal anti-inflammatory drugs (NSAIDs), acetaminophen, and multimodal pain management plans shows that these approaches decrease the overall amount of opioids as long as used adequately for pain management [8]. However, these options suggest that opioids cannot be eliminated in patients with high pain tolerance and those who fail to receive sufficient relief from other drugs. The literature thus highlights a dual perspective: Short-term use of opioids is beneficial for example in the first few hours after surgery, while long-term use comes with severe adverse effects. It is, therefore, imperative to develop patient-centred discharge models that address opioid education or even monitoring to enhance outcomes and mitigate risks related to strong opioids in post-discharge care [13].

Our research aims at assessing postoperative pain relief and satisfaction of strong opioids in discharged patients with lower-limb large joint arthroplasty, in regard to patient-perceived pain, satisfaction, and risk factors.

## Materials and methods

This retrospective cross-sectional study was conducted at the arthroplasty ward in the Trauma and Orthopaedics Department of Russells Hall Hospital, Dudley Group NHS Foundation Trust, a district general hospital in Dudley, West Midlands, in the United Kingdom from January 1, 2024 to January 31, 2024.

The study commenced after receiving approval from the ethical review committee of the hospital (approval no. QI/2023-24/03). Patients meeting the inclusion criteria were recruited from the ward. Patients aged 18-60 years, both genders, who underwent lower-limb large joint replacements including total hip replacement (THR) and total knee replacement (TKR) were included. Patients having allergies to any pain medications and prolonged hospital stays of more than four days in the hospital were excluded. Informed and written consent was obtained from each patient prior to enrollment, with the details of the study thoroughly explained to them.

A non-probability consecutive sampling technique was used. A total of 50 patients were enrolled in the study, out of which 25 had THR and 25 had TKR. The collection included demographics, pain medications upon discharge letters, and pain satisfaction scores. The patients were further divided into two groups with Group A having paracetamol, NSAIDs, and weak opioids upon discharge, whereas the other group, named Group B, was given strong opioids upon discharge.

The patients had a telephonic follow-up on the 28th postoperative day (POD). This follow-up included their initial five days of pain experience at home and they were asked to rate that pain as per the universal pain assessment tool. Pain score was further divided into four major categories that are no pain at all, mild pain (score 1-3), moderate pain (score 4-6), and severe pain (score 7-10). All these data were then compiled on Microsoft Excel (Microsoft Corp., USA), and data analysis was done on IBM SPSS Statistics for Windows, Version 23.0 (released 2015, IBM Corp., Armonk, NY), with a p-value of ≤0.05 considered statistically significant.

## Results

In this study, examining the role of strong opioids in effective discharge management for lower-limb large joint arthroplasty patients, the demographic and clinical characteristics of two patient groups were compared (Table 1). Group A, comprising patients discharged with strong opioids, and Group B, discharged with weak opioids, showed no significant difference in mean age (36.4 ± 13.5 for Group A and 36.48 ± 10.6 for Group B; p = 1.0). Gender distribution was also similar, with males representing 52% in Group A and 48% in Group B and females representing 48% in Group A and 52% in Group B (p = 0.327). Regarding the type of large joint replacement, there was no significant difference between the two groups; 32% of Group A and 36% of Group B underwent TKR, while 68% of Group A and 64% of Group B had THR (p = 0.327). 

**Table 1:** Clinical and demographic parameters of the study participants THR: total hip replacement, TKR: total knee replacement, POD: postoperative day

Parameters	Group A (strong opioids)	Group B (weak opioids)	P-value
Mean age	36.4±13.5	36.48±10.6	1.0
Gender			0.327
Male	13 (52%)	12 (48%)	
Female	12 (48%)	13 (52%)	
Large joint replacement			0.327
TKR	8 (32%)	9 (36%)	
THR	17 (68%)	16 (64%)	
Pain score on 5th POD			0.001
No pain	6 (24%)	3 (12%)	
Mild pain (score 1-3)	11 (44%)	11 (44%)	
Moderate pain (score 4-6)	6 (24%)	5 (20%)	
Severe pain (score 7-10)	2 (8%)	6 (24%)	

However, a significant difference in pain scores on the fifth POD was observed between the two groups (p = 0.001). Group A reported higher instances of no pain (24%) compared to Group B (12%), while both groups had similar rates of mild pain (score 1-3) at 44%. Moderate pain (score 4-6) was reported by 24% of Group A and 20% of Group B. Severe pain (score 7-10) was more common in Group B (24%) than in Group A (8%). 

**Figure 1:**
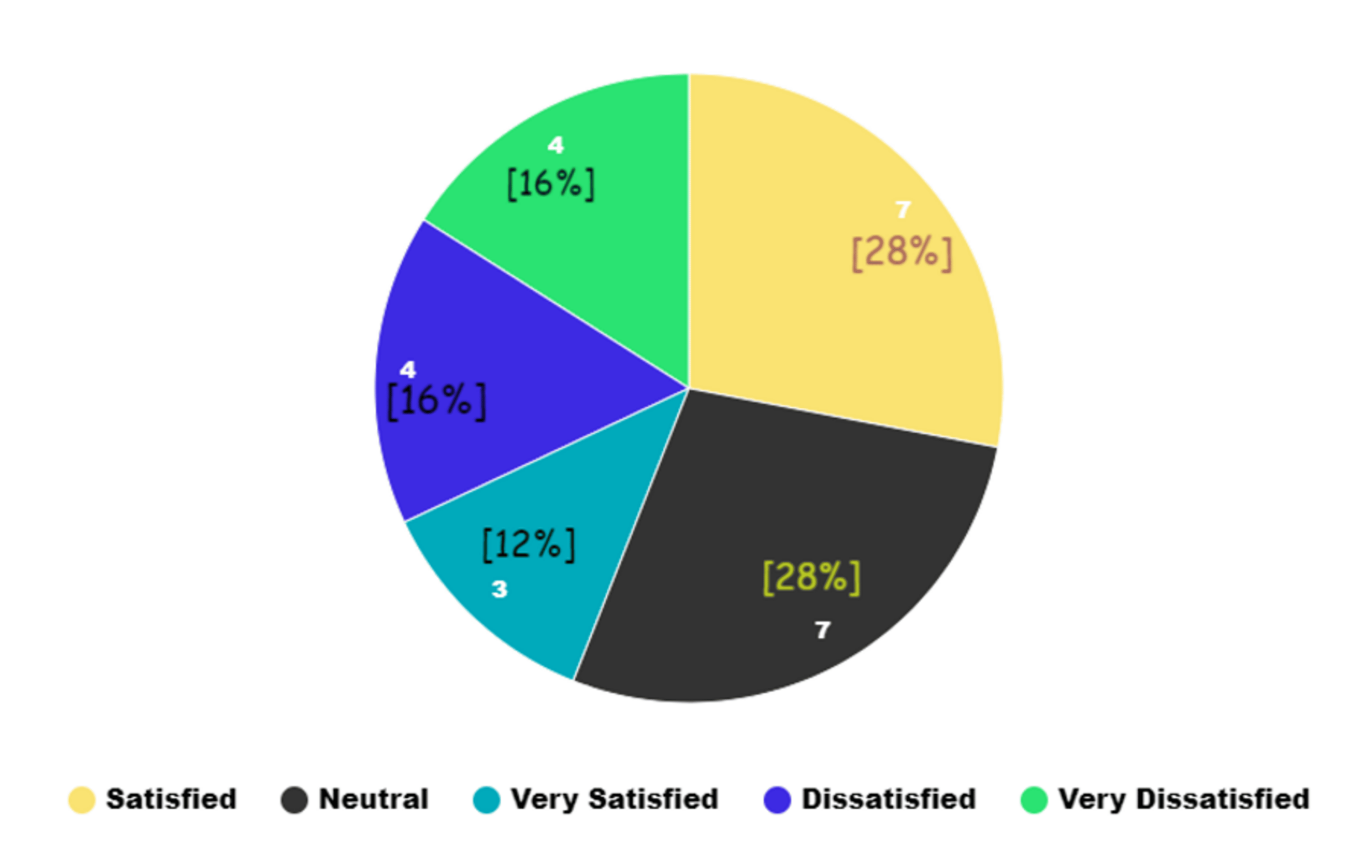
Patient satisfaction score in Group A

**Figure 2:**
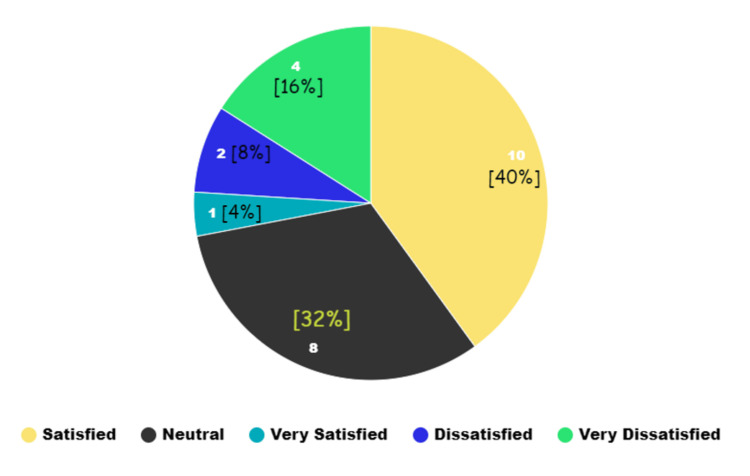
Patient satisfaction score in Group B

Patient satisfaction levels also differed notably between the groups (Figures 1, 2), with significant variance (p = 0.011). Group A showed a higher rate of "very satisfied" patients (n = 3) compared to Group B (n = 1). Satisfaction levels showed seven patients in Group A and 10 in Group B feeling "satisfied," while seven in Group A and eight in Group B reported feeling "neutral." Dissatisfaction was slightly higher in Group A (four patients "dissatisfied" and four "very dissatisfied") compared to Group B, which had two "dissatisfied" and four "very dissatisfied" patients. 

## Discussion

Effective pain management in the early postoperative period after lower-limb large joint arthroplasty is crucial, as inadequate pain control can hinder mobilisation, delay recovery, and reduce patient satisfaction [14]. Adequate analgesia is crucial especially in the first few PODs in order to encourage mobilisation, shorten hospital stays, and enhance patient satisfaction [15]. For procedures such as THR and TKR, pain may be influenced by the surgical approach, patient characteristics, and the use of analgesic agents [16]. Literature review indicates that individualized pain management that consists of a combination of non-pharmacological therapies and pharmacological interventions with the use of strong opioids occasionally is feasible for achieving pain relief [17,18]. Nonetheless, to achieve optimum effectiveness, the side effect-patient preference trade-off remains a major concern post-surgery [19]. 

In our department, the postoperative analgesic plan is in accordance with the ERAS protocol that strictly avoids the use of opioids and instead combines several types of pain medications. This involves the use of paracetamol, NSAIDs, and either weak opioids or strong opioids depending on the patient’s requirement. Our approach is therefore consistent with the ERAS guidelines, specifically regarding the sparing use of opioids and the administration of these where required in the smallest effective concentrations. Recent research on ERAS-based practices suggests that effective pain management and minimization of opioid side effects are achievable with ERAS protocols. For instance, a number of studies have shown that ERAS protocols where non-opioid analgesics and local anaesthesia are used provide the same level of pain relief as traditional opioid-based regimens with fewer negative impacts [20]. However, other trials implementing non-ERAS standardized protocols demonstrate higher rates of opioid consumption after hospital discharge and longer postoperative length of stay together with lower global satisfaction, thus underlining the advantage of the multimodal approach in the management of pain in the perioperative period [21]. 

In our study, pain scores on the fifth POD were significantly lower in patients discharged with strong opioids (Group A) compared to those discharged with weak opioids (Group B). For instance, while only 8% of the Group A patients complained of severe pain after the intervention, 24% of the Group B participants made the same complaint. This is consistent with the results of other works that report a decrease in pain intensity under the influence of strong opioids, especially during the first days after surgery. However, due to side effects and its addictive nature, many institutions have sought other or additional measures of pain management. For example, Haskel et al. (2020) compared pain management using strong opioids with that of weaker opioids or non-opioid analgesics post-THA: patients with strong opioids had significantly lower pain scores in the first week after discharge. However, the study also found that it is a bitter fact that patients taking strong opioids suffer from the side effects of opioids and received mixed overall satisfaction studies though maintained satisfactory pain control [22]. Likewise, Frassanito et al. (2020) conducted a study on TKR in which strong opioids prescribed during discharge were associated with lower pain scores but more side effects like urinary retention and constipation, which reduced the over-recovery satisfaction [23]. Liu et al. (2019) compared ERAS protocol patients with those on traditional opioid-centered pain management, finding that the ERAS approach, which focuses on multimodal analgesia, resulted in lower pain scores and fewer side effects. They reported comparing an opioid-dominant regime that achieved a mean five-day postoperative pain score of 2·3 with a modified ERAS regime, which was less opioid-based and focused on multimodal analgesia using NSAIDs and local anaesthetic infiltration, which provided equal pain relief but without the side effects of opioids [24]. 

In this study, patient satisfaction levels were affected by the type of opioids prescribed at the time of discharge. Despite the negative responses concerning side effects, most of the patients belonging to Group A, which was using strong opioids, had recorded higher levels of satisfaction and there were records of dissatisfaction emanating from opioid side effects. This is in concordance with other numerous studies that have clearly shown that despite being effective for initial acute pain, strong opioids might not necessarily offer satisfying results for patients because of the potential side effects they can develop. For instance, a cross-sectional from Karam et al. (2021) regarding pain control after TKA showed that patients who were discharged on strong opioids had relatively better pain control in the early mobilization phase and were more likely to report postoperative satisfaction regarding pain control. However, the same studies observed that due to the usual side effects of opioids like nausea, dizziness, and constipation, patient dissatisfaction also increased during the same process of recovery [25]. The same pattern of higher satisfaction for good pain control and lower satisfaction due to side effects is reported in other similar studies and is meaningful despite acknowledging that the side effects of opioids are multifaceted in patients’ perceived surgical care. 

Limitations

This is a retrospective study with a small sample size. Moreover, we did not look into the correlation of pain score with comorbidities, which decreases pain threshold. In future, we aim to do a prospective study with a larger sample size so that the results could be applied to the population. 

## Conclusions

Our study brings attention to a highly individualized approach to multimodal pain management using strong opioids for large joint arthroplasty patients that provide high-quality pain management and patient satisfaction. However, there exist certain problems with increasing the supple strength of analgesics and their side effects. Thus, there also exists the requirement of carrying out further research regarding the multimodal regimens that will offer effective and superior pain control to the patients. In future practice, we propose the extension of the ERAS protocol-based opioid-inclusive regimes and subsequent inclusion of, for example, extended-release local anaesthetic or nerve blocks. Furthermore, discharge analgesia should also be planned according to the patient’s evaluation so that the recovery results are satisfactory. 
